# Sulphate of Cinchonia

**Published:** 1859-05

**Authors:** Robert Battey

**Affiliations:** Rome, Georgia


### ARTICLE II.

*Sulphate of Cinchonia.*—By ROBERT BATTEY, M.D., Rome, Ga.

Two years' experience in the use of this salt as a partial substitute for sulphate of quinine, has impressed me favorably as to its merit, and leads me now to adopt it entirely for the future in my practice. The following are the conclusions which appear to me to be legitimately drawn from my field of observation.

1st. There is no desirable effect from the administration of quinia, which is not equally obtainable from cinchonia. 2d. The undesirable effects of our doses of quinia, comprehended in the term *quininism*, have not been shown to result from large and repeated doses of cinchonia. 3d. It is entirely practicable to administer the cinchonia to patients who cannot bear even moderate doses of quinia. 4th. A freedom from the apprehension of quininism enables the practitioner to exhibit the cinchonia in frequent and large doses, and secure effects which could not have been obtained so satisfactorily from quinia. 5th. As a tonic it is more acceptable to the stomach than quinia. 5th. Though bitter to the taste it is not as intensely so as quinia. 7th. *Five* grains of the sulphate of cinchouia are equivalent in antiperiodic power to *four* grains sulphate of quinia. 8th. The price of the cinchonia salt, about one-third that of quinia, commends it for hospital use and for country physicians.

It is not my purpose to present any one of these propositions as a fully demonstrated fact; I desire only to offer them as deductions chiefly from my own observations in the use of the remedies.

Upon the first item, I deem it unnecessary to cite cases in support of the position taken. I have used the cinchonia and quinia in cases (for the most part remittent and intermittent fevers and neuralgia,) as nearly parallel as practice would afford, and treated perhaps an equal number with each. In some instances I have given ten grains at two hours for three successive doses, have used it in many cases where moderate and even quite small doses of quinine are badly borne; and in no instance have I observed any approach to quininism, or indeed any unpleasant effect to contra-indicate its use. Whether still larger doses of cinchonia be capable of producing this undesirable condition, I am not prepared either to affirm or dispute, having never found it desirable to exceed the thirty grains in the space of four hours. It is with me a favorite tonic, and particularly in chronic diseases of the uterus. My usual formulæ are—Sulphate of cinchonia, 2 or 4 drachms; sulphuric acid, 10 drops; simple syrup, a pint—mix and direct a teaspoonful three times daily.——Sulphate of cinchonia, 2 or 4 drachms; elixir vitriol, 2 ounces; simple syrup, a pint—mix, and direct as above.——Sulphate of cinchonia, 4 drachms; muriated tincture of iron, 2 oz.; simple syrup, a pint—mix, and direct as above. This salt is entirely compatible with syrup, of iodide of iron, and the two may be combined in the following elegant preparation: Sulphate of cinchonia, 1 dr'm; citric acid, 30 grains; simple syrup, 3 oz.—mix, make a solution, and add: syrup of iodide of iron, 1 oz.—mix, and direct a teaspoonful three times daily.

The alkaloid (cinchonia) I have found a preferable form for administering the remedy to children; its slow solubility rendering it much less bitter to the taste.

